# Effectiveness of engaging religious leaders in maternal health education for improving maternal health service utilization in Ethiopia: cluster randomized controlled trial

**DOI:** 10.3389/fpubh.2024.1399472

**Published:** 2024-07-29

**Authors:** Abinet Arega Sadore, Yohannes Kebede, Zewdie Birhanu

**Affiliations:** ^1^Department of Health, Behaviour, and Society, Faculty of Public Health, Institute of Health, Jimma University, Jimma, Ethiopia; ^2^School of Public Health, College of Medicine and Health Sciences, Wachemo University, Hosaina, Ethiopia

**Keywords:** religious leaders, maternal health, health education, health promotion, Ethiopia

## Abstract

**Introduction:**

High mortality rates for pregnant women and their new-borns are one of Africa’s most intractable public health issues today, and Ethiopia is one of the countries most afflicted. Behavioral interventions are needed to increase maternal health service utilizations to improve outcomes. Hence, this trial aimed to evaluate effectiveness of trained religious leaders’ engagement in maternal health education on maternal health service utilization.

**Methods:**

The study employed a cluster-randomized controlled community trial that included baseline and end-line measurements. Data on end points were gathered from 593 pregnant mothers, comprising 292 and 301 individuals in the intervention and control groups, respectively. In the intervention group, the trained religious leaders delivered the behavioral change education on maternal health based on intervention protocol. Unlike the other group, the control group only received regular maternal health information and no additional training from religious leaders. Binary generalized estimating equation regression analysis adjusted for baseline factors were used to test effects of the intervention on maternal health service utilization.

**Results:**

Following the trial’s implementation, the proportion of optimal antenatal care in the intervention arm increased by 21.4% from the baseline (50.90 vs. 72.3, *p* ≤ 0.001) and the proportion of institutional delivery in the intervention group increased by 20% from the baseline (46.1% vs. 66.1%, *p* ≤ 0.001). Pregnant mothers in the intervention group significantly showed an increase of proportion of PNC by 22.3% from baseline (26% vs. 48.3%, *p* ≤ 0.001). A statistically significant difference was observed between in ANC4 (AOR = 2.09, 95% CI: 1.69, 2.57), institutional delivery (AOR = 2.36, 95% CI: 1.94, 2.87) and postnatal care service utilization (AOR = 2.26, 95% CI: 1.79, 2.85) between the intervention and control groups.

**Conclusion:**

This research indicated that involving religious leaders who have received training in maternal health education led to positive outcomes in enhancing the utilization of maternal health services. Leveraging the influential position of these religious leaders could be an effective strategy for improving maternal health service utilization. Consequently, promoting maternal health education through religious leaders is advisable to enhance maternal health service utilization.

**Clinical trial registration:** [https://clinicaltrials.gov/], identifier [NCT05716178].

## Background

Maternal mortality is unacceptably high in the World. About 287,000 women died during and following pregnancy and childbirth in 2020. Almost 95% of all maternal deaths occurred in low and lower middle-income countries in 2020, and most could have been prevented (1).

Many developing countries face challenges that make it difficult for pregnant women and new-borns to get the healthcare they need. People are not aware of the services, their cultures and traditions make them hesitant, their families do not always support them, they prefer to do things at home, transportation and costs can be an issue, and religious or traditional leaders may oppose them (2–4).

Each year, approximately 112,000 newborns and 14,000 mothers in Ethiopia die due to preventable reasons (5). In Ethiopia, there are high rates of maternal deaths and poor health outcomes for women, which could be due to the fact that too many women are not using the available maternal health services like antenatal, skilled birth, and postnatal care (6, 7). Having access to health facilities is a big factor in how healthy and safe a mother is during her pregnancy and childbirth, especially for women living in places with limited resources (8).

Involving families, religious leaders, and traditional authorities in maternal health initiatives is a powerful strategy. Religious organizations can be leveraged to raise awareness and educate communities about available services. Their trusted position and respect within communities make them ideal partners in bridging the gap to better maternal and neonatal healthcare (9). Similarly, research indicates that religious leaders, medical specialists, and healthcare personnel have a significant impact on people’s beliefs, feelings, and behaviors (10). Religious leaders have the power to influence people’s health habits on all levels, from individual to global, which can have a huge effect on the health of a community. This is done through providing health education and helping people make healthy choices (10, 11). The influence of faith leaders on health habits aligns with the Ottawa Charter for Health Promotion’s emphasis on community involvement. This is because faith leaders can mobilize their communities to participate in health initiatives (11). Communities need to be given the power to take part in and manage their own matters. A religious leader, as a vital member of the community, is in a great position to inspire others to modify their behavior. Reports on programs that involve faith-based groups show that clergy members were successful in helping bring about changes in behavior, especially with people who are hard to reach (10, 12). Other research has highlighted the significance of faith leader’s effect on health habits (13). Similarly, Christian religious leader’s impact on behavior has been associated with Bible-based teachings that support healthy living (10, 14). While some studies suggest spiritual leaders can impact their congregants’ health behaviors, the strength and specific methods of this influence remain under-investigated (15).

Religious organizations can reach a large audience due to their places of worship. Plus, religious leaders are often leaders in the community and are the people who provide people with information. They have the potential to increase awareness regarding underused resources for maternal and new-born health (16). Some Africans have reported trust in religious-based leadership, rather than political leadership. Sub-Saharan Africa, a region with a huge unmet need for FP, has examples of faith leaders acting as family planning advocates (17). There has not been any research done to specifically look at how trained religious leaders’ participation in maternal health education affects maternal health service utilization in Ethiopia, even though they may act as change agents and influence others to improve maternal health service utilization and health behaviors during pregnancy. This cluster-randomized trial was conducted to assess how the involvement of trained religious leaders in maternal health education impacts the utilization of maternal health services.

## Methods

### Study setting

The research study took place in two districts within the Hadiya Zone: Lemo and Ameka. The Hadiya Zone is located approximately 232 kilometers away from Addis Ababa, the capital city of Ethiopia. It falls within the Southern Ethiopia Regional State. The Hadiya Zone Health Department report for 2019–2020 states that about 1,650,104 people were living in the zone overall, with 830,002 of them being female. The Hadiya Zone has a network of healthcare facilities including 311 health posts, 60 rural health centers, and four hospitals (18). All pregnant women living in Hadiya Zone’s rural areas were considered as source populations. However, the study only included pregnant mothers living in specifically selected clusters during the research period (study population). The study was conducted from February 2023 to September 2023.

### Study population

Ethiopia’s society is multi-religious, with a sizable Muslim population in addition to the majority Christian population. Of Ethiopia’s population, around two-thirds (62.8%) are Christians, and one-third (34%), Muslims. Those who practice traditional African religions and other faiths make up a tiny portion of the populace (19). This study included pregnant women <20 weeks gestational age, lived in the selected study area for at least 6 months and able to communicate. Exclusion criteria: Pregnant women who were diseased or unable to provide data at the time of data collection were excluded.

### Study design

A cluster-randomized controlled trial single-blind parallel-group, two-arm trials with a 1:1 allocation ratio was conducted among pregnant mothers. The trial was carried out in compliance with Consolidated Standards of Reporting Trials (CONSORT) guidelines for cluster-randomized studies (20). Community settings that promote group participation were used to administer the intervention. To reduce intervention contamination and maximize logistical convenience, the randomization unit was clusters, or kebeles (small administrative units).

#### Sample size determination

According to the EDHS 2019, skilled birth attendance in a rural area of Ethiopia was predicted to be 43% (21). The estimated sample size was designed to detect variations in the utilization of skilled delivery services, which aligns with the main objective of the broader study. Based on the following assumptions, the sample size was calculated using Stata software. Tail (s): 1; effect size (d): 0.15; power (1-β error probability) = 0.8; α error probability = 0.05; and N1/N2 allocation ratio = 1. The resultant sample size was 155. Then, adding design effect, DE = 1 + (m^−1^) ICC, where *m* = Average cluster size, *s* = 50, ICC = 0.02 based on a published study ICC (22), DE = 2. The overall sample size was 682 after accounting for a 10% follow-up loss (N1 = 341 and N2 = 341). The sample sizes for the control and intervention groups are N1 and N2, respectively. A sample size of 341 pregnant mothers per group was determined based on these assumptions, to detect a 15% point predicted difference in the proportion of skilled delivery service utilization (an increase from 43 to 58%).

### Randomization

**Figure 1:**
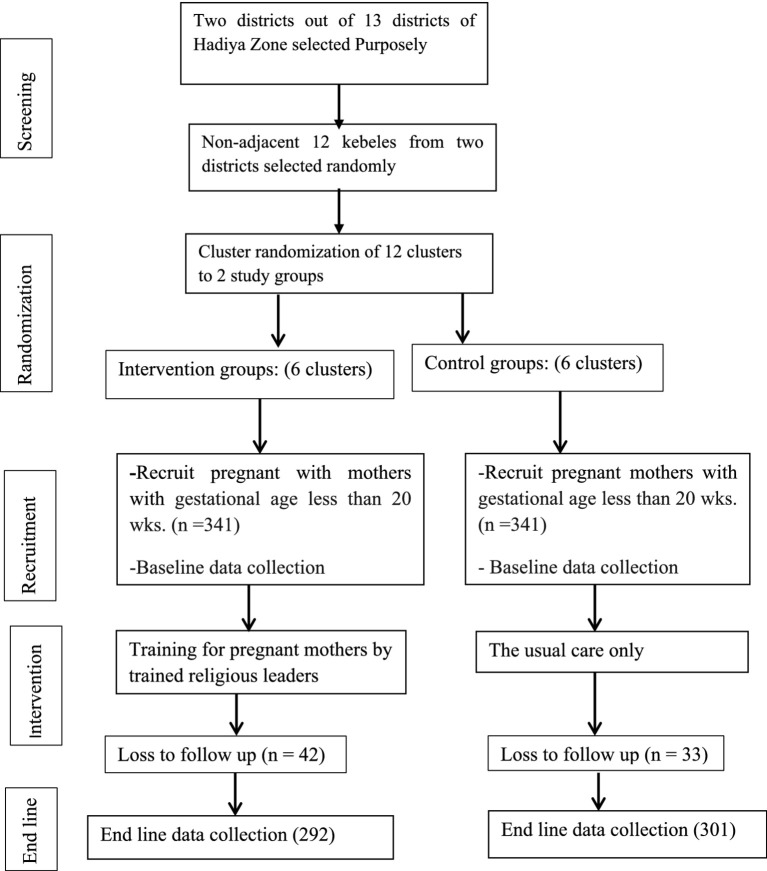
Diagrammatic representation of the randomization of study cluster (kebeles).

The approach of cluster sampling in two stages was employed. First, two districts were selected purposely out of 13 rural districts in the Hadiya Zone. Second, the districts’ administrative offices provided listings of every cluster in the chosen districts. Samples of nonadjacent kebeles (clusters) from the two districts were selected using a simple random sampling technique. Six clusters out of 14 clusters from the Ameka district and six clusters out of 16 clusters from the Lemo district were selected based on their proportion to size allocation and considering cluster allocation in the intervention and control groups. Clusters were assigned to control or intervention groups using simple randomization with a 1:1 allocation. The final units of observation were pregnant mothers within each cluster. The results were reported following the CONSORT guidelines (Figure 1). Religious leaders provided training on maternal health to the intervention group, while the control group was left to continue the existing services.

## Recruitment

Women from the study areas who met the inclusion criteria were recruited. All pregnant women who satisfied the inclusion requirements were included in the study. Health extension personnel selected and screened the study participants. Pregnant women who satisfied the requirements were screened by the first day of their last menstrual cycle as part of a house-to-house survey. Subsequently, an human chorionic gonadotropin (HCG) urine test was employed to verify the pregnancy in these mothers. HCG levels in urine as well as urine-based pregnancy tests were employed. During the process, a test strip was dipped into a urine sample. The test strip contained substances known as monoclonal antibodies, which reacted to the presence of the pregnancy hormone HCG. On the test strip, the outcomes are shown as lines.

### Concealment

The cluster allocation was concealed from data collectors by not disclosing it to them, excluding them from the trial implementers, and not residing in any of the clusters. During enrolment and the gathering of baseline data, trial participants were kept blind to the existence of an intervention. Data collectors, religious leaders, and participants were all provided with no information about the study’s hypothesis. Supervisors on the field were not aware of the outcome of the investigation. Nevertheless, it was not feasible to blind the religious leaders and trial participants who were receiving the intervention because of the design and nature of the intervention being studied. To maintain data integrity and impartiality, data collectors were unaware of the intervention allocation for each cluster and did not reside in any of the participating clusters. However, due to the nature of the intervention, trial participants were aware of the group to which they had been assigned.

### Intervention

While pregnant women in the control group did not receive any maternal health training intervention, they did receive maternal health education during routine services and any community-based interventions by health extension workers. Religious leaders with training in maternal health provided four months of training to the pregnant mothers in the intervention group. During the intervention period, Hadiyisa, the local language, was spoken. The intervention comprised three distinct parts.

### Part 1: recruiting and training of religious leaders

The study involved religious communities in the Hadiya Zone. Initially, relevant faith-based organizations within the designated study areas were identified. Then, local leaders representing these communities were chosen based on their religious training (minimum diploma level), their standing and influence within their communities, and the endorsement of community leaders, healthcare workers, and local authorities. Sixteen religious leaders were selected to receive training aimed at improving maternal health outcomes. The principal investigator led a four-day training designed to equip these leaders with the knowledge, attitude, and communication skills needed to effectively support and encourage pregnant women to utilize recommended healthcare services. The training combined theoretical sessions and practical demonstrations, ensuring alignment with the intervention protocol and community acceptance. Religious leaders were equipped with the necessary skills and resources to deliver culturally appropriate training sessions for pregnant women. These training sessions were conducted in the local language and utilized facilitator manuals and posters to enhance understanding and engagement. A variety of engaging and interactive teaching methods were employed to impart knowledge on maternal health to religious leaders. These methods included talks, group discussions, group work exercises, demonstrations, role-plays, storytelling, simulations, case studies, and problem-solving. The trained religious leaders were tasked with conducting four sessions on maternal health topics for their congregations, with the aim of enhancing the use of maternal health services among their members.

### Part 2: training of pregnant mothers

For four months, trial subjects in the interventional group received behavioral change intervention on maternal health, whereas the control group’s pregnant mothers received standard care. In collaboration with health extension workers, kebeles’ leaders and religious leaders, researchers, and field workers, appropriate training places were arranged in the groups of intervention to educate pregnant women about maternal health. The trained religious leaders conducted four group training sessions for pregnant mothers, adhering to the same training procedures they had received during their own training. The first training session took place at the outset of the intervention, followed by a second session with the same content one month later. The third and fourth training sessions were conducted one and two months after the second session, respectively. Following the training sessions, participants received visual materials (posters) summarizing Key ideas for encouraging behaviors that promote maternal health. The training sessions employed direct, interactive, and participatory learner and activity-oriented instructional strategies. These strategies included talks, group discussions, group work exercises, demonstrations, role-plays, storytelling, simulations, case studies, and problem-solving, all aimed at enhancing knowledge, attitude, and behaviors related to maternal health.

### Part 3: home visit

In addition to group training sessions, in the intervention group, religious leaders made four home visits to each pregnant mother. During these home visits, religious leaders provided personalized counseling and support on maternal health, adhering to a structured counseling protocol. These home visits served as a reinforcement mechanism, helping pregnant mothers translate the knowledge and skills gained from group training sessions into positive maternal health behaviors.

### Key intervention messages

1. Essential practices of antenatal care like tetanus toxoid injections, calcium and iron and folic acid tablet intake, healthy eating, and HIV/syphilis testing.
2. Recognizing danger signs during pregnancy, labor, postpartum, and neonatal stages.
3. Consulting healthcare professionals for danger signs.
4. Birth preparedness and complication readness.
5. Early and exclusive breastfeeding.
6. Debunking unhealthy beliefs.
7. Proper hygiene for maternal and child well-being.

### Monitoring and quality control measures

A process assessment was conducted to closely look at how the intervention was implemented, ensuring it was delivered as intended by using yes or no questions. This assessment examined how well religious leaders engaged in the intervention and the effectiveness of reaching the intended audience, specifically pregnant mothers. The intervention activities were assessed whether they were implemented as planned by the number of training sessions held with religious leaders, the number of visual materials distributed to religious leaders, and the number of visual materials distributed to pregnant mothers. The measurement of the intervention’s success in reaching the targeted pregnant mothers was based on the quantity of recruited and trained pregnant mothers.

### Data collection and management

The lead researcher provided training to the data collectors and supervisors over a span of two consecutive days. To capture baseline information, a standardized interviewer-administered questionnaire was administered to enrolled pregnant mothers in both study groups simultaneously. This questionnaire assessed maternal and household characteristics. Following the conclusion of the intervention period, simultaneous end-line data collection was carried out for both the intervention and control groups to assess the utilization of maternal health services. Data collection occurred at five-month intervals to ensure robust longitudinal data. The data type was repeated cross-sectional. Experienced data collectors with a background in healthcare and proficiency in the local language were recruited to collect the data. Additionally, a supervisor with a master’s degree in a healthcare profession was employed to oversee the entire data collection process.

### Measurements and indicators

Questionnaires contained sections on socio-demographics, Reproductive history of the respondent, maternal health service utilizations. The prevalence of antenatal care (ANC) utilization was evaluated based on two criteria: “at least one ANC attendance” refers to women who have participated in a minimum of one ANC check-up during their current pregnancy. “Four or more ANC utilization” pertains to women who have attended four or more ANC visits throughout their current pregnancy. These criteria were determined based on self-reported data provided by the participants (23). The evaluation of safe delivery service utilization involved identifying women who delivered their babies in a health center or hospital, receiving care from skilled healthcare professionals such as midwives, nurses, health officers, and doctors. This information was obtained through self-reported data provided by the participants (24). Postnatal care service utilization was assessed by determining whether women had utilized postnatal care services at least once within 42 days after childbirth. This included postnatal care visits at health facilities for women who had given birth at home, as well as post-discharge follow-ups for women who gave birth in a healthcare facility (25).

### Data quality control

To guarantee the quality of data, data collectors and supervisors received training, and supervisors and principal investigators conducted regular monitoring and follow-up activities. In addition, data integrity and consistency were checked regularly on a daily basis. The questionnaire was initially translated into the local language, Hadiyisa, and subsequently into English by a translator who was not familiar with the original questionnaire. Pilot test and pre-testing of the tool were conducted in areas with similar characteristics to the study population to ensure clarity, wording, logical order, and abbreviated patterns of questions. Pregnant women who underwent pre-testing were not incorporated into the study, and adjustments were implemented.

The intervention’s fidelity was upheld in accordance with the best practice recommendations established by the National Institutes of Health Behavioral Change Consortium (26). The intervention design incorporated a conceptual framework, and non-adjacent clusters were chosen to avoid the risk of information contamination. An equal number of clusters were selected from each district for both the intervention and control groups to ensure balance and mitigate variations. To ensure consistency and effectiveness, the intervention process was thoroughly tested before the trial’s main implementation. Every pregnant mother underwent an equal number of training sessions with four months durations, ensuring fair access to the intervention. Group education training followed a standardized format, utilizing a training manual, role-playing activities, and mock counseling practice to enhance learning and engagement. The competency of recruited religious leaders was monitored by a pre-post training test. The test focused on knowledge and attitudes toward intervention key messages. If the post-training test score of the trained religious leaders was below the acceptable range, retraining would be given on identified gaps of intervention messages. A select number of training sessions were randomly chosen for process evaluation, with each selected session being assessed by a single process evaluator. The process evaluator rated the trainer using a “yes/no” rating system on various aspects, including adherence to the training guide, delivery of the entire content, adherence to the training duration and frequency, preparation, accuracy, and ability to adequately respond to questions. To assess the reception of the intervention, interviews were conducted with pregnant mothers to evaluate their understanding of the key content related to maternal health. The implementation of the intervention was measured by examining the rates of utilization for antenatal care, skilled delivery, and postnatal care.

### Statistical analysis

The data entry process included double entry into EPI-info software, and subsequently, the data was exported to SPSS version 25 for both data cleaning and statistical analysis. Descriptive statistics were utilized to display the baseline characteristics of the participants in the study. McNemar’s test was employed to assess the proportions of the outcome variables in both the intervention and control groups. Additionally, differences before and after the intervention were measured to compare changes between the intervention and control groups. The Generalized Estimating Equation (GEE) with a binary logit function was applied to examine variations in the change of outcomes between the intervention and control groups. The GEE accommodates clustered data and correlation of observations within subjects. During model fitting, the independent covariance matrix was considered. The odds ratio (OR) with a 95% confidence interval (CI) was calculated as the outcome measure for assessing the impact of the intervention.

## Results

From the initial 668 pregnant women enrolled in the study, 593 (89%) were ultimately included in the final analysis. This comprised 292 participants in the intervention group and 301 participants in the control group. The remaining 21 pregnant women in the intervention group discontinued participation due to various reasons: 16 failed to attend all of the religious leader-led training sessions, and five decided not to participate due to scheduling conflicts. Likewise, 10 pregnant women relocated, three experienced miscarriages, seven faced illness, and 13 declined participation in the study. Thirty-three pregnant women in the control group were lost to follow-up (Figure 1).

### Baseline characteristics

At the commencement of the study, there were no significant distinctions in socio-demographic and obstetric characteristics between the intervention and control groups, suggesting an equitable distribution across both groups (*p* > 0.05) (Table 1).

**Table 1:** The baseline characteristics of study population, Hadiya Zone, Southern Ethiopia.

Variable	Category	Intervention group (*n* = 334)	Control group (*n* = 334)	*p*-value
Number of clusters		6	6	
Age	15–24 25–34 35–49	78(23.4) 221(66.2) 35(10.5)	71(21.3) 218(65.3) 45(13.5)	0.130
Religion	Protestant Orthodox Muslim Catholic	210(62.9) 114(34.1) 4(1.2) 6(1.8)	208 (62.3) 88 (26.3) 24 (7.2) 14(4.2)	0.241
Maternal education	No formal education Primary education (1–8) Secondary education (9–12) Tertiary education (college or university)	26(7.8) 177(53.0) 108(32.3) 23(6.9)	30(9.0) 194(58.1) 91(27.2) 19(5.7)	0.172
Husband education	No formal education Primary education (1–8) Secondary education (9–12) Tertiary education (college or university)	9(2.7) 141(42.2) 140(41.9) 44(13.2)	9(2.7) 169 (50.6) 126(37.7) 30(9.0)	0.301
Mothers’ occupation	Housewife Merchant Government employee Farmer	272(81.4) 3(0.9) 18(5.4) 41(12.3)	278(83.2) 4(1.2) 17(5.1) 35(10.5)	0.697
Family size	1–2 persons 3–4 persons ≥ 5 persons	36(10.8) 137(41.0) 161(48.2)	41(12.3) 148(44.3) 145(43.4)	0.443
Planning pregnancy	Planned Unplanned	278(83.2) 56(16.8)	295(88.3) 39(11.7)	0.178
Gravidity	Primigravida Multigravida	130(38.9) 204(61.1)	117(35) 217(65)	0.338
Parity	Nullipara Primipara Multipara	115(34.4) 78(23.4) 141(42.2)	132(39.5) 84(25.1) 118(35.3)	0.293

### Baseline maternal healthcare service utilization

Prior to the commencement of the trial, there were no statistically significant disparities between the two groups regarding their overall utilization of antenatal care, institutional delivery, and postnatal care services (Table 2).

**Table 2:** Baseline data of the utilization of maternal health services in the Hadiya Zone, Southern Ethiopia 2023.

Variables	Intervention	Control	*P*-value
ANC visit (1–2 times)	(*n* = 334)	(*n* = 334)	0.417
Yes	170 (50.9%)	179 (53.6%)	
No	164 (49.1%)	155 (46.4%)	
Institutional delivery	(*n* = 219)	(*n* = 202)	0.375
Yes	101 (46.1%)	95 (47%)	
No	118 (53.9%)	107 (53%)	
Previous PNC (at least once)	(*n* = 219)	(*n* = 202)	0.417
Yes	57 (26%)	48 (23.8%)	
No	162 (74%)	154 (76.2%)	

### The difference between baseline and end-line maternal health service utilization and difference of the differences between intervention and control groups

Examination of the intervention group indicated a notable rise of 21.4% in the percentage of women accessing antenatal care services. This positive change was statistically significant (*p* < 0.001 a). Conversely, the control group observed a slight 1.9% upturn in the utilization of antenatal care services. The difference-in-difference in antenatal care service utilization between the two groups was 19.5%, signifying a notable impact of the intervention (*p* < 0.001 a). A similar pattern was observed for institutional delivery services. The intervention group demonstrated a significant 20% rise in institutional delivery rates, and this difference was statistically significant (*p* < 0.001 a). On the contrary, the control group witnessed a marginal 1.8% decline in institutional delivery rates. The difference-in-difference in the utilization of institutional delivery services between the two groups was 21.8%, offering further support for the effectiveness of the intervention (*p* < 0.001 a). Ultimately, the intervention group demonstrated a notable 22.3% rise in the utilization of postnatal care services in comparison to the control group. The difference-in-difference in the utilization of postnatal care services was 16.9%, emphasizing the favorable influence of the intervention on this facet of maternal health (*p* < 0.001 a) (Table 3).

**Table 3:** The variation in percentage of maternal health service utlization in the intervention and control groups in Hadiya Zone, Southern Ethiopia.

Variables	Control			Intervention			
	Baseline	End line	Difference (EL-BL)^a^	Baseline	End line	Difference (EL-BL)^a^	Difference of difference^b^
ANC4+	53.6	55.5	1.9	50.9	72.3	21.4	19.5**
Institutional delivery	47.0	45.2	−1.8	46.1	66.1	20.0	21.8**
PNC (at least once)	23.8	29.2	5.4	26	48.3	22.3	16.9**

BL, baseline; EL, end line. ^a^The difference was calculated by subtracting end-line values from baseline values. ^b^The difference of differences was calculated by subtracting the difference in the controls from the difference in the intervention groups. ^**^p < 0.001.

### Generalized estimating equation intervention results on maternal health service utilization

As per the Generalized estimating equation (GEE) model, pregnant women in the intervention group were 2.09 times more inclined to receive optimal antenatal care compared to their counterparts in the control group (AOR = 2.09, 95% CI: 1.69, 2.57). Similarly, the intervention group showed a 2.36-fold increase in institutional delivery rates compared to the control group (AOR = 2.36, 95% CI: 1.94, 2.87). The intervention’s favorable effect also reached postnatal care utilization, as the intervention group demonstrated a 2.26 times higher likelihood of utilizing postnatal care services compared to the control group (AOR = 2.26, 95% CI: 1.79, 2.85) (Table 4).

**Table 4:** GEE results on the influence of the involvement of religious leaders in maternal education on the utilization of maternal health services in Hadiya Zone, Southern Ethiopia.

Variables	Study group	*N*(%)	Beta coefficient	Standard error	AOR(CI)	*p*-value
ANC	CG	167(55.5)			1	
	IG	211(72.3)	0.37	0.152	2.09(1.69, 2.57)	0.000
Institutional delivery	CG	136(45.2)			1	
	IG	193(66.1)	0.861	0.098	2.36(1.94, 2.87)	0.000
PNC	CG	88(29.2)			1	
	IG	141(48.3)	0.815	0.118	2.26(1.79, 2.85)	0.000

CG, control group (*n* = 301); IG, intervention group (*n* = 292); AOR, adjusted odd ratio; CI, confidence interval. AOR were yielded by using GEE (Generalized estimated equation) regression analysis.

## Discussion

Religious institutions reach a wide audience through their places of worship, and religious leaders often serve as community leaders who disseminate information. They can play a vital role in promoting awareness about underutilized maternal and neonatal health services. Nevertheless, there is insufficient evidence regarding the effectiveness of maternal health education led by religious leaders in enhancing maternal health service utilization among pregnant women, particularly in rural areas. This study utilized a community-based cluster-randomized controlled trial design to assess the impact of involving trained religious leaders in maternal health education on the improvement of maternal health service utilization. The primary focus of the intervention was to enhance the utilization of maternal health services in rural areas. The intervention was designed based on findings from the baseline formative phase, which encompassed qualitative and quantitative studies. Therefore, this behavioral intervention was based on a context-specific target population. At the study’s outset, the socio-demographic features, obstetric characteristics, and maternal health service utilization of pregnant women were comparable.

In this study, the intervention had a notable impact on the utilization of maternal health services, indicating that the involvement of religious leaders in maternal health education can enhance the utilization of such services. Following the trial’s implementation, optimal ANC service utilization improved by 19.5% compared to control group in which intervention not occurred, which was estimated based on the intervention group baseline and control group baseline and end line study. The intervention had a significant impact on increasing the number of optimal ANC visits, which we believe is consistent with a mechanism of action involving increased recognition of the importance of completing the full course of recommended ANC visits, leading to greater motivation to participate in ANC. Therefore, we believe that this intervention was particularly effective in motivating pregnant women to complete all of their ANC visits. Maternal health education led by trained religious leaders resulted in an improvement in facility deliveries compared to the baseline group. The difference-in-difference analysis indicates that the intervention led to a 21.8% increase in the intervention group. We noticed an increase in the utilization of ANC4 and PNC in the control group during the intervention period compared to the baseline period. In contrast, there was a decrease in institutional delivery in the control group during the intervention period. The results of our study support previous research showing that religious leaders were effectively involved in maternal health (27). Our intervention, involving the engagement of religious leaders in maternal education alongside existing services, enhances the utilization of ANC4, institutional delivery, and postnatal care services. A systematic review uncovered indications that mHealth interventions could raise attendance at ANC4 visits by approximately 10%, in contrast to the 19.5% increase observed in the present study (28). A single community-based intervention appeared to have a slight positive impact on institutional births, attendance of at least ANC1 visits, and attendance of at least ANC4 visits, according to a systematic review and meta-analysis. This was supported by other studies that tested the impact of working with religious leaders to promote maternal health (27, 29). The findings of this study can be applied to Ethiopian Muslims, Orthodox Muslims, and protestant Muslims. We think that the intervention might work in different contexts.

The majority of disclosures of religious leaders’ involvement in mother and child health promotion initiatives have been successful in promoting family planning, particularly in Muslim-majority nations (30, 31). In this study, it was noted that engaging religious leaders in health promotion activities in rural areas posed challenges. Some noted that they require fuel and vehicles to enable them reach community gatherings; however, in most cases, they do not have the means. These information was gathered from trained religious leaders and participants in the intervention group during process evaluation of the intervention implementation.

Studies showed that community members complained that religious leaders were talking about family planning inappropriately and that they were straying from their mission of preaching the word of God. Myths and misconceptions about contraception, religious hostility to family planning. Religious leaders were unable to answer technical queries from family planning clients or potential clients (32).

The general consensus among the religious leaders was that attitudes toward these maternal and child health, sexual and reproductive health, and gender equality messages often varied between older and younger people and also between men and women (33). Because religious leaders are sought by their communities for advice on almost every aspect of daily life, including reproductive health issues, they need scientific and updated information, which prepares them to correct misconceptions, dispel rumors, and provide useful advice (34).

A particular challenge for partnerships between secular development organizations and religious organizations is the perception that religious organizations are weak in key areas of global health implementation, especially program management, monitoring and evaluation (35). Advocating for family planning with religious leaders necessitated far more relationship development and a keen understanding of terminology (30).

The findings of this research work have important policy implications. Given the importance of religion in Ethiopia’s sociocultural fabric and the position of influence, authority, and respect occupied by various religious leaders (i.e., pastors, evangelists, priests, imams, and sheiks), one way of achieving a rapid increase in Ethiopia’s use of maternal health services may be continuously engaging religious leaders at all levels in advocacy efforts. Religious leaders wield great influence over their followers. Their body language and messages can inhibit or facilitate effective health care-seeking behaviors. Studies showed religious belief as a key determinant of maternal healthcare provision utilizations in Ethiopia (6, 36). It is anticipated that the use of maternal health services will rise if religious leaders are involved in maternal health education. The study unveiled that religion exerts a significant influence on women’s health. For instance, the persistence of religious opposition to contraceptive adoption in Ethiopia is attributed, in part, to the dissemination of myths and misconceptions about family planning (37, 38). Hence, collaborating with religious leaders emerges as a critical strategy to enhance maternal health service utilization in Ethiopia. The study’s results emphasize the significance of involving religious leaders in initiatives aimed at improving maternal health service utilization in Ethiopia. This suggests that the engagement of religious leaders in maternal health education, when executed effectively alongside other reproductive health activities, could yield meaningful outcomes not only in Ethiopia but also in other Sub-Saharan African countries facing similar challenges.

### Strengths and limitations of the study

The difference of difference analysis enabled us to estimate the intervention effect compared to the expected counterfactual outcome had the intervention not occurred. This study’s robustness stems from its randomized controlled trial design and a relatively large sample size. However, some limitations exist. Firstly, the study’s nature precluded a double-blind design. Second, providing the intervention for 6 months, maybe a short time to bring a huge change in maternal health service utilizations, and third, as the study was based on self-reported data, respondents may have answered questions with the intention of satisfying the interviewer, thus adding social desirability bias to the results despite data collectors arranged a comfortable environment by keeping mothers apart and making them free during data collection.

## Conclusion

This trial underscores the potential effectiveness of engaging trained religious leaders in maternal health education to improve the utilization of maternal health services in rural areas. Engaging religious leaders as change agents in maternal health promotion activities is crucial for achieving effective outcomes, as evidenced by this trial. The observed findings in this experiment are suggestive of the important role of trained religious leaders in the improvement of maternal health service utilization. We are confident that the approach of collaborating with religious leaders to enhance maternal health service utilization can be adapted to various settings in different regions and countries. Further study of the applicability of this intervention is needed.

## Data availability statement

The original contributions presented in the study are included in the article/supplementary material, further inquiries can be directed to the corresponding author.

## Ethics statement

The studies involving humans were approved by the Institutional Review Board of Jimma University. The studies were conducted in accordance with the local legislation and institutional requirements. Written informed consent for participation in this study was provided by the participants' legal guardians/next of kin.

## Author contributions

AS: Writing – review & editing, Writing – original draft, Software, Resources, Project administration, Methodology, Investigation, Funding acquisition, Formal analysis, Data curation, Conceptualization. YK: Writing – review & editing, Writing – original draft, Visualization, Validation, Supervision, Resources, Project administration, Methodology, Investigation, Conceptualization. ZB: Writing – review & editing, Writing – original draft, Validation, Supervision, Resources, Project administration, Methodology, Investigation, Funding acquisition, Conceptualization.
